# UK case control study of smoking and risk of amyotrophic lateral sclerosis

**DOI:** 10.1080/21678421.2019.1706580

**Published:** 2020-04-17

**Authors:** Sarah Opie-Martin, Ashley Jones, Alfredo Iacoangeli, Ahmad Al-Khleifat, Mohamed Oumar, Pamela J. Shaw, Chris E. Shaw, Karen E. Morrison, Robyn E. Wootton, George Davey-Smith, Neil Pearce, Ammar Al-Chalabi

**Affiliations:** 1 Department of Basic and Clinical Neuroscience, Maurice Wohl Clinical Neuroscience Institute, King’s College London, London, United Kingdom,; 2 Sheffield Institute for Translational Neuroscience (SITraN), University of Sheffield, Sheffield, United Kingdom,; 3 Faculty of Medicine, University of Southampton, Southampton, United Kingdom,; 4 MRC Integrative Epidemiology Unit at the University of Bristol, University of Bristol, Bristol, United Kingdom,; 5 School of Psychological Science, University of Bristol, Bristol, United Kingdom,; 6 NIHR Bristol Biomedical Research Centre, University Hospitals Bristol NHS Foundation Trust and University of Bristol, Bristol, United Kingdom,; 7 London School of Hygiene and Tropical Medicine, London, United Kingdom

**Keywords:** Amyotrophic lateral sclerosis, smoking, case control

## Abstract

*Introduction*: Susceptibility to amyotrophic lateral sclerosis (ALS) is associated with smoking in some studies, but it is not clear which aspect of smoking behavior is related. Using detailed records of lifetime smoking we investigated the relationship between smoking and ALS in a UK population. *Methods*: In this retrospective case-control study, smoking status was collected using environmental questionnaires from people diagnosed with ALS between 2008 and 2013 and from age, sex and geographically matched controls. Categorical measures of smoking behavior were: smoking at the time of survey and smoking initiation; continuous measures were intensity (cigarettes per day), duration (years from starting to stopping or time of survey), cigarette pack years, and comprehensive smoking index (CSI), a measure of lifetime smoking. We used logistic regression to assess the risk of ALS with different combinations of smoking variables adjusted for age at survey, gender, level of education, smoking status and alcohol initiation, selecting the best model using the Akaike Information Criterion. *Results*: There were 388 records with full smoking history. The best-fitting model used CSI and smoking status at the time of survey. We found a weak association between current smoking and risk of ALS, OR 3.63 (95% CI 1.02–13.9) *p* value 0.05. Increase in CSI score did not increase risk of ALS: OR 0.81 (95% CI 0.58–1.11) *p* value 0.2.*Conclusion*: There is weak evidence of a positive effect of current smoking on the risk of ALS which does not show dose-dependence with higher levels of lifetime smoking and maybe a false positive result.

## Introduction

Amyotrophic lateral sclerosis (ALS) is a neurodegenerative disease characterized by progressive death of motor neurons leading to relentlessly worsening weakness and death, usually from respiratory failure due to involvement of the diaphragm, 2–3 years after diagnosis (1,2) Although there is an evident genetic component, heritability studies indicate that environmental (and probably stochastic) factors also contribute (3–6).

There is evidence from multiple studies that smoking is associated with ALS, but no agreement over which aspect of smoking behavior is related to ALS (7–14). Despite an evidence-based literature review that concluded that smoking can be considered a risk factor for ALS, it remains unclear if there is a dose-response effect, or what the biological mechanism might be (15). In addition, confounding cannot be discounted, since ALS is also associated with military service, education and socioeconomic status, which are also associated with smoking status (16,17). It is biologically plausible that smoking could be a risk factor through oxidative stress or exposure to potentially neurotoxic chemicals, and so it remains an attractive candidate for studies of environmental etiology (18,19).

The comprehensive smoking index (CSI) estimates lifetime smoking by combining duration, intensity and time since cessation into a score allowing all factors to be considered while avoiding issues of multicollinearity between smoking exposure variables (20). CSI has not previously been used to investigate the role of smoking in ALS risk.

We, therefore, analyzed retrospective case-control data to determine whether smoking is related to ALS in a UK population, investigating the relationship between different smoking variables including CSI and other regularly used measures, and risk of ALS.

## Methods

### Case-control study design

The data were obtained from the Motor Neurone Disease Association of England, Wales and Northern Ireland (MNDA) Collections collected as part of the MNDA Epidemiology Study, REC reference 07/MRE01/57. People diagnosed with definite, probable or possible ALS according to the El Escorial criteria between 2008 and 2013 were included (21). Three tertiary centers in London, Sheffield, and Birmingham acted as data collection hubs but people with ALS were recruited at secondary centers such as district general hospitals, therefore these are incident cases representative of the ALS population. General practitioners from the general practice of the person with ALS were asked to invite 10 healthy controls to participate in the study via post. The research team matched people on age (within 5 years of the person with ALS) and gender in a 1:1 ratio. Four hundred and thirteen participants provided informed consent, 405 undertook a telephone interview about their lifestyle including smoking undertaken by a trained nurse. Three participants gave no information on smoking behavior.

### Definition of smoking status

Categorical measures were: smoking at the time of survey (current, former, never), smoking initiation (ever, never).

To define former smokers we used logistic regression modeling to compare ALS risk between current smokers and ex-smokers, using never smokers as a reference. Few people had recently quit (*n* = 3 within one year of survey) so we grouped ex-smokers into 5-year time since cessation intervals up to 20 years which was aggregated to 20+. ALS risk reduced from an odds ratio of 2.02–0.79 for current smokers compared to people who had quit within 5 years so former smokers were defined as having given up at least a day before the survey.

Continuous measures included: intensity (cigarettes per day), duration of smoking (years from starting to stopping or time of survey), pack years (intensity × duration), and CSI. The CSI is a non-linear model of smoking exposure that combines duration of smoking, time since cessation and smoking intensity into a continuous score which can be used in a regression model representing lifetime smoking (20). The model involves the simulation of tau and delta from the dataset. Delta, or half-life, reflects the exponential decay in the effect of smoking on health outcomes during a lifetime. Tau, or lag-time, reflects that smokers may be at a higher risk of disease immediately after quitting due to reverse causality. The equations for CSI are as follows:

tsc*=max(tsc−δ, 0)

dur*=max(dur+tsc−δ, 0)–tsc*

$$\text{comprehensive smoking index}=(1–0.5^{\text{dur}*/\tau })\;(0.5^{\text{tsc}*/\tau })\;\ln\left(\mathrm{int}+1\right)$$

tsc: time since cessation; *δ*: lag time; tss: time started smoking; dur: duration of smoking (calculated as age-tss for people currently smoking or [age-tsc]-tss for former smokers); *τ*: half-life; int: cigarettes per day.

### Logistic regression

Data were analyzed using R (22). Continuous demographic characteristics were compared by Student’s *t*-test or Mann-Whitney *U* test. Categorical variables were compared by chi-squared or Fisher’s exact test. The primary outcome, whether smoking increases the risk of ALS, was analyzed using logistic regression with maximum likelihood estimation. We generated eight models with combinations of one categorical and one continuous measure of smoking, comparing the Akaike Information Criterion (AIC) of the models to assess fit (23). Odds ratios were adjusted for age, educational attainment, gender, and alcohol consumption.

Assuming an odds ratio of 1.8, a 20% smoking rate in the control population and alpha of 0.05, we had 71% power with a sample size of 400 cases and controls in a 1:1 ratio.

## Results

There were 202 cases and 200 control records available for analysis. The two groups were similar except for educational attainment and alcohol status. The details are shown in Table 1.

**Table 1:** Unadjusted comparisons of demographics and behavior in ALS cases and controls.

Demographic/behavioral measure	Case (*n* = 202)	Control (*n* = 200)	*p* Value (test)
Gender ratio, Female: Male % (*n*)	41:59 (85:117)	44:56 (88:112)	0.77 (Chi-squared test)
Educational attainment % (*n*)			
Primary school	1.5 (3)	1 (2)	0.0041 (Fisher’s exact test)
Secondary school	38.1 (77)	30.5 (61)	
College	31.2 (63)	23.5 (47)	
Technical school	8.4 (17)	12 (24)	
University	14.4 (29)	29 (58)	
Other	5.5 (12)	3.5 (7)	
Missing	0.5 (1)	0.5 (1)	Not analyzed
Mean age at survey (standard deviation)	63.1 (10.53)	64.5 (10.52)	0.12 (*t*-test)
Alcohol use % (*n*)			
Alcohol status	8:62 (17:184)	12:88	0.32
Never: Ever		(24:176)	(Fisher’s exact test)
Site of onset % (*n*)			
Bulbar	21.7 (44)	n/a	
Spinal	73.3 (148)	n/a	
Not known/recorded	5 (10)	n/a	
Mean age at onset (SD)	60.7 (10.6)	n/a	
Median months onset–diagnosis (IQR)	12 (13)	n/a	
Median months onset– survey (IQR)	28.1 (21.5)	n/a	

The three centers are tertiary referral centers with about a third of the patients diagnosed at the center, and the remainder diagnosed elsewhere first. SD: standard deviation; IQR: interquartile range; n/a: not applicable.

The optimal CSI variables were tau = 2 and delta = 3.6. There were no differences between groups in unadjusted smoking behaviors, as shown in Table 2.

**Table 2:** Smoking variables and crude comparisons.

Smoking measure	Case	Control	*p* Value
Smoking behavior % (*n*)			
Smoking initiation (ever smokers)	47 (94)	53 (105)	0.27
Smoking status	47:44:9	53:44:3	0.065
Never : Former : Current	(94:90:18)	(105:88:7)	
Median age smoking initiation (*n*)	16 ± 3 (108)	16 ± 3.5(94)	0.94
Median cigarettes per day (*n*)	17 ± 10	15 ± 10	0.88
Median duration smoking	23.5 ± 25.6	23 ± 24	0.58
Median cigarette pack years	20 ± 27.27	16 ± 28.4	0.7
Median comprehensive smoking index values	0.031 ± 1.85	0.0053 ± 1.36	0.33

Chi squared tests were used for categorical variables and Mann-Whitney *U* for continuous variables as all were non-normally distributed. Six people were missing duration information, 4 missing smoking intensity. Records with missing data were excluded from analysis.

Three hundred eighty-eight records had full smoking history available for logistic regression analysis. Table 3 gives the results of the best fitting logistic regression model which included the CSI and smoking status at time of survey with AIC 543.77. The highest AIC, representing the worst fitting model, was for smoking initiation and number of cigarettes per day at 553.23. An increase in the value of CSI did not increase the risk of ALS: OR 0.81 (95% CI 0.57–1.11) *p* value 0.2. Current smoking increased the risk of ALS, OR 3.62 (95% CI 1.02–13.9) *p* value = 0.05, a Bonferroni correction shows that this is likely a false positive result because of multiple testing.

**Figure 1:**
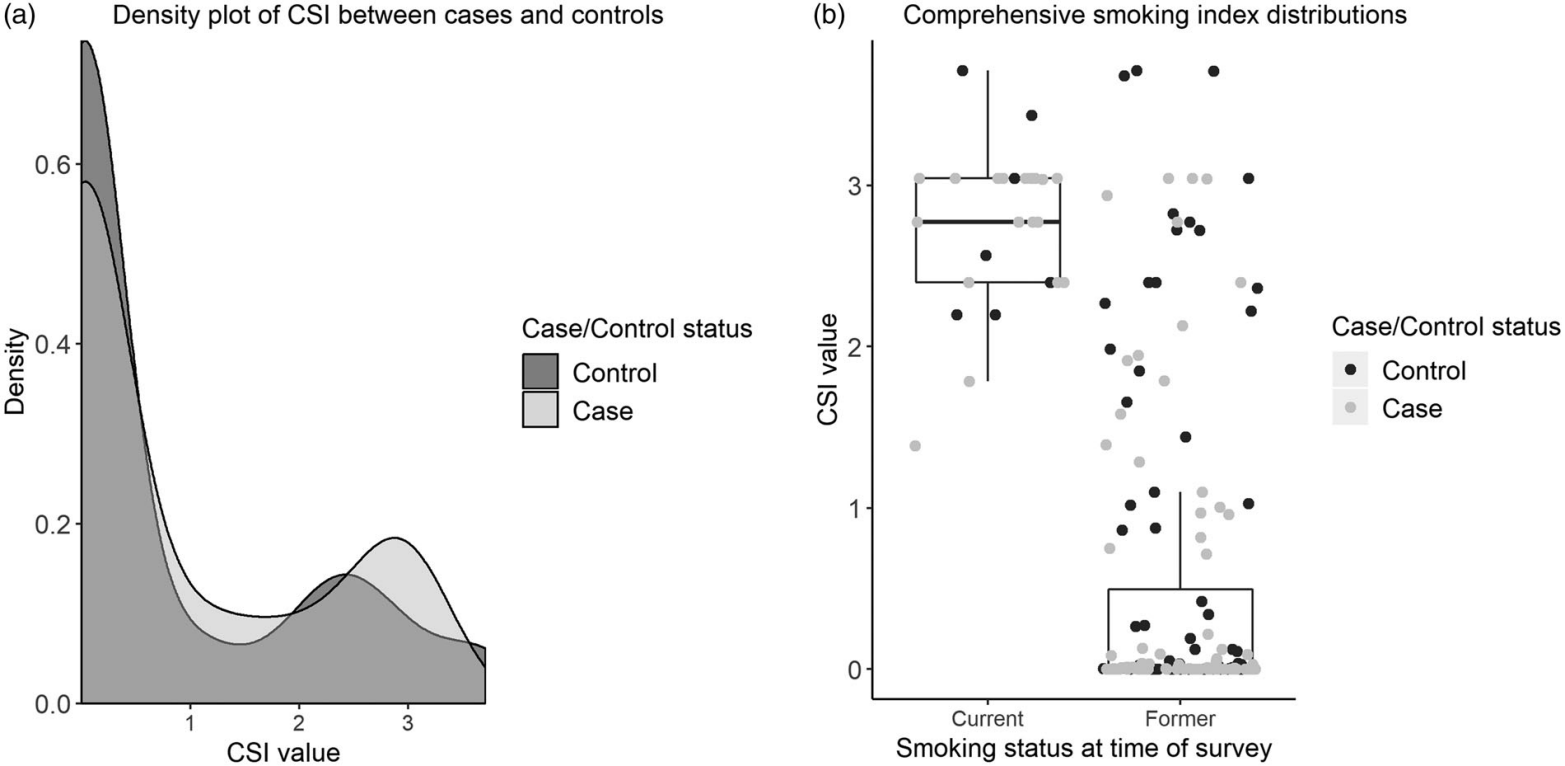
(a) Density plot of CSI value by case control status. (b) Box plot of CSI value by smoking status at time of survey, points colored by case control status. Both graphs are in ever smokers only.

**Table 3:** Best fitting logistic regression model for smoking and risk of ALS.

Variable	Odds ratio	Lower CI	Upper CI	*p* Value
Smoking status				
Current smoker	3.62	1.02	13.8	0.05
Former smoker	1.08	0.67	1.74	0.74
Comprehensive smoking index	0.81	0.58	1.11	0.2
Age	0.98	0.96	1	0.07
Ever drinker	1.33	0.65	2.75	0.43
Male	1.05	0.68	1.63	0.83
Education level				
Primary school	2	0.05	78.4	0.68
Secondary school	1.27	0.05	33.2	0.87
Technical school	0.73	0.03	19.4	0.83
College	1.32	0.05	34.4	0.85
University	0.44	0.02	11.7	0.58
Other	1.6	0.06	45.7	0.75

## Discussion

We found a weak association between current smoking and risk of ALS using traditional epidemiology methods to explore association. We report an uncorrected *p*-value of 0.05, and several models tested for fit, suggesting that this is, in fact, a false-positive result. We also found that using CSI to measure lifetime smoking exposure resulted in a better fitting model for our data than using cigarette pack years but we found no evidence of a dose-dependent response of ALS risk to smoking.

Our results are similar to those from a study conducted in the Netherlands which found current smoking to be associated with ALS in an incident cohort but no strong dose-dependent relationship (9). The strength of the association between smoking and ALS was reported as weak in a meta-analysis of case-control and cohort studies, with a higher effect in women (7). This weakness may be due to the reliance on prevalent and clinic cohorts which would under-represent smokers because their survival is shorter (9).

A pooled analysis of prospective studies found that there was an increased risk of ALS in former and current smokers (13). Two large prospective cohort studies included in the pooled analysis were originally set up as prospective studies into environmental exposures and cancer risk (11,14). People with ALS were identified from death certificates, which may over-represent people who smoke as their survival is shorter.

The CSI is more useful than cigarette pack years to investigate dose-dependency, as it formally considers the decreased risk of disease after smoking cessation. The CSI had a bimodal distribution of smoking exposure in both cases and controls, corresponding to smoking at the time of survey (Figure 1). The mean CSI of current smokers is slightly higher in cases than controls and so dose-dependency in current smokers should be investigated further.

Median age of smoking initiation was around the late teens in both groups, and it has been reported that frontotemporal dementia, a behavioral change that occurs in some people with ALS is not associated with smoking behaviors, so the association is unlikely to reflect reverse causality (24).

The strengths of this study are that we have detailed environmental data on incident cases of ALS and controls. A limitation is the sample size which means it is only powered to detect relatively large effect sizes with odds ratios of the order of 1.8 or higher. Retrospective case-control studies generally suffer from recall bias. This study may suffer the effect of two opposing sample biases: people in an environmental study of lifestyle may be more likely to smoke heavily, and some people in this ALS study attended specialist clinics so may be less likely to smoke. Additionally, we do not know how many controls who were contacted declined to participate, so the control population may be biased. There were no current smokers in the controls recruited in London, although a subgroup analysis in the other two areas shows that odds ratios for current smoking are consistent between the remaining areas.

We found that people with ALS were less likely to drink alcohol, but our survey responses do not support a protective relationship as ALS was cited as the reason for not drinking in most cases. Despite controlling for drinking and educational status, it is not possible to completely rule out the effects of confounding.

In this study of smoking and ALS, we do not find strong evidence to support smoking as a risk factor, even using lifetime smoking exposure as measured by the CSI.
